# Genome-wide association analysis of stripe rust resistance loci in wheat accessions from southwestern China

**DOI:** 10.1007/s13353-019-00533-8

**Published:** 2020-01-07

**Authors:** Bin Cheng, Xu Gao, Ning Cao, Yanqing Ding, Yu Gao, Tianqing Chen, Zhihai Xin, Liyi Zhang

**Affiliations:** 1 Insititute of Upland Crops, Guizhou Academy of Agricultural Sciences, Guiyang, 550006 Guizhou China; 2 College of Agriculture, Guizhou University, Guiyang, 550025 Guizhou China; 3 College of Life Sciences, Guizhou University, Guiyang, 550025 Guizhou China

**Keywords:** Stripe rust, *Yr* genes, Population structure, Southwestern China, Illumina 90K SNP array

## Abstract

Wheat stripe rust can cause considerable yield losses, and genetic resistance is the most effective approach for controlling the disease. To identify the genomic regions responsible for *Puccinia striiformis* f. sp. *tritici* (*Pst*) resistance in a set of winter wheat strains mainly from southwestern China, and to identify DNA markers in these regions, we carried out a genome-wide association study (GWAS) of 120 China winter wheat accessions using single nucleotide polymorphism (SNP) markers from 90K wheat SNP arrays. In total, 16 SNP loci were significantly associated with wheat stripe rust in field and greenhouse trials. Of these, three distinctive SNPs on chromosomes 1B, 4A, and 6A were identified at a site in Mianyang in 2014, where the most prevalent wheat stripe rust races since 2009 have been V26 (G22-9, G22-14). This suggests that the three SNP loci were linked to the new quantitative trait loci (QTL)/genes resistant to the V26 races. Germplasm with immunity to *Pst* is a good source of stripe rust resistance for breeding, and after further validation, SNPs closely linked to resistance QTLs/genes could be converted into user-friendly markers and facilitate marker-assisted selection to improve wheat stripe rust resistance.

**Electronic supplementary material:**

The online version of this article (10.1007/s13353-019-00533-8) contains supplementary material, which is available to authorized users.

## Introduction

Stripe rust, caused by *Puccinia striiformis* f. sp. *tritici* (*Pst*), is one of the most destructive fungal diseases, causing significant grain yield losses under severe infections in wheat fields where high levels of moisture (Solh et al. 2012). It seems a big challenge to increase wheat yields to satisfy the growing global populations, because the denser plant canopies of wheat cultivars under high nitrogen fertilizer supply favor the development of stripe rust. China is one of the severest epidemic areas in the world. The disease frequently occurs in winter wheat growing areas in the Northwest, Southwest, and North China (Wan et al. 2004; Wellings 2011). Using resistant varieties is an effective way to control wheat stripe rust.

Wheat stripe rust resistance is affected by both race-specific and non-race-specific resistance genes. Until now, approximately 83 officially designed (*Yr1–78*) and 47 temporarily named stripe rust resistance genes have been identified in wheat and its wild relatives (McIntosh et al. 2014; Maccaferri et al. 2015; McIntosh et al. 2017). These resistance genes have been mapped to various chromosomes. However, many genes no longer show disease resistance due to the rapid mutation of the *Pst* pathogen. Currently, only a few of these genes have been used in actual production in China, such as *Yr9*, *Yr10*, *Yr18*, *Yr26*, etc. In 2009, a new pathogen of wheat stripe rust was identified in Sichuan, called V26 (or G22 pathogens) because it is virulent in wheat cultivars carrying the *Yr26* gene. A recent study found that V26 is also highly virulent to the majority of *Yr* genes, except for *Yr5*, *Yr15*, and *Yr61* (Zeng et al. 2015). The V26 pathogen has now spread to several major wheat-producing areas and constitutes a great threat to wheat production in China. Therefore, it is essential to discover new genes for stripe rust resistance and incorporate them into elite wheat germplasms to protect them against this devastating disease.

As a supplement to traditional quantitative trait loci (QTL) mapping, genome-wide association studies (GWAS) detect statistical associations between phenotypic and genetic variations throughout the genome. With the development of the wheat 9K and 90K single nucleotide polymorphism (SNP) iSelect array technique, GWAS has been used increasingly to identify and dissect resistance genes or QTLs in common wheat. Using the 9K SNP iSelect array, Zegeye et al. (2014) identified 27 and 38 SNPs associated with resistance to *Pst* at the seedling and adult plant stage in 181 synthetic hexaploid wheat, respectively; Maccaferri et al. (2015) analyzed a worldwide collection of 1000 spring wheat accessions and identified 97 loci that are significantly associated with *Yr* resistance in at least three environments, of which 3 likely represent new resistance loci. Naruoka et al. (2015) discovered potentially novel QTLs associated with race-specific seedlings and adult plant responses on chromosomes 1B, 1D and 2A, 2B, 3B, 4A, and 4B, respectively. Jighly et al. (2015) used a strategy, combining Diversity Arrays Technology (DArT®) with 9K SNP assays in 200 ICARDA wheat genotypes, 12 DArT and 29 SNP markers were identified to significantly associate with *Pst*-resistance genes. Liu et al. (2017) identified 68 SNP loci that are associated with seedling resistance to one or more races in 182 durum wheat landraces from Ethiopia. Muleta et al. (2017a) found that 15 SNP loci in Ethiopian germplasm are significantly associated with seedling and adult plant resistance (APR) to stripe rust, where 3 loci on chromosomes 5A and 7B may be novel. In 959 spring wheat varieties, the same researchers found 11 and 7 chromosome regions that significantly associated with stripe rust resistance at the adult plant and seedling stages, respectively (Muleta et al. 2017b).

In this study, we analyzed a set of 120 common wheat landraces mainly from Southwest China to evaluate their seedling stage and adult plant responses to stripe rust infection in multiple environments. We carried out GWAS based on the 90K SNP iSelect array. Our objectives were (1) to determine which genes or loci are associated with stripe rust resistance in this panel and (2) to identify SNP loci awaiting further exploitation in marker-assisted selection.

## Materials and methods

### Plant materials

The 120 wheat (*Triticum aestivum* L.) cultivars (lines) included in our study represent diverse cultivars that are cultivated in winter wheat regions of Southwest China (Supplementary Table S1). Among these, 63 accessions were derived from Guizhou province, 34 from Sichuan Province, and 23 from other regions in China.

### DNA extraction, genotyping, and known *Yr* gene detection

Genomic DNA was extracted using the cetyl trimethyl ammonium bromide method (Saghai-Maroof et al. 1984). Wheat accessions were genotyped using the wheat 90K SNP iSelect assay developed by a consortium of laboratories from the USA, UK, and Australia and published in Wang et al. (2014). The assay was conducted by Compass Biotechnology Co., Ltd. (Beijing, China). SNP genotypes were called using GenomeStudio v2011.1 software (Illumina Inc.). The Illumina 90K SNP array yielded 81,587 SNPs; of these, 164 (0.2%) could not be assigned to known map positions. SNPs with minor allele frequencies of less than 5% or with more than 20% of data missing were removed.

According to the chromosomes of significant SNP identified in our GWAS, seven markers were selected to scan known *Yr* gene loci in our collection (Supplementary Table S2), in order to investigate the correlation among these gene loci and significant SNPs. The PCR reaction was carried out in an ABI Thermal Cycler in a volume of 20 μl containing 1.0 U Taq DNA polymerase, 5 mM KCl, 1 mM Tris-HCl, 0.15 mM MgCl_2_, 200 μM of each dNTP, 0.5 μM of each primer, and 80–100 ng of template DNA. The PCR reactions were as follows: denaturation at 94 °C for 4 min, followed by 35 cycles of 94 °C for 1 min, 50–60 °C (depending on primers) for 1 min, 72 °C for 1 min, and a final extension for 10 min at 72 °C. PCR products were mixed with 4 μl of the formamide loading buffer (98% formamide, 10 mM EDTA, 0.25% bromophenol blue, 0.25% xylene cynol, pH 8.0) and heated at 94 °C for 5 min. Each sample of 5 μl was loaded on 6% denaturing polyacrylamide gels and run at 100 W for about 2 h and then resolved by the silver staining method as described by Bassam et al. (1991).

### Disease evaluations

#### Stripe rust response in the fields

A total of 120 accessions were evaluated under natural pathogen infestation conditions in four field trials, which were carried out at three locations (Guiyang and Hezhang in 2013, Guiyang and Mianyang in 2014). The wheat lines Avocet S and Mingxian 169 were used as the susceptible control. Eighty seeds of each line were sown in 60-cm-wide paired-row plots, 1.2 m in length, with 30-cm row spacing and a 50-cm pathway between plots. Disease severity (DS) was classified as 0, 1%, 5%, 10%, 25%, 40%, 65%, and 100% throughout the season. We evaluated the adult plant stage, which is when the stripe rust reaction of the susceptible Mingxian169 cultivar was most severe. Infection types (ITs) 0-0, 1, 2, 3, and 4 were characterized as immune (IM), nearly immune (NIM), highly resistant (HR), moderately resistant (MR), moderately susceptible (MS), and highly susceptible (HS), respectively. Cultivars with the ITs of 0-0, 1, or 2 were classified as resistant, while those with the ITs 3 or 4 were classified as susceptible.

#### Stripe rust tests in the greenhouse

Seedlings of the 120 accessions were evaluated for IT response to *Pst* races (CRY32, CRY33) under controlled greenhouse conditions. The *Pst* races were maintained at the State Key Laboratory of Crop Stress Biology for Arid Areas, Northwest Agriculture & Forestry University (China). Ten seeds of each accession were planted in a plastic seedling-raising plate (540 × 280 × 80 mm, 72 holes) and grown in a growth chamber. Seedlings at the two-leaf stage (14 days after planting) were inoculated separately with urediniospores of each *Pst*. The inoculations were carried out by brushing conidia of isolates onto the seedlings to be tested, when the first leaf was fully expanded. Inoculated seedlings were then placed in plastic-covered cages and incubated at a temperature of 10 °C and relative humidity (RH) of 100% for 24 h. The seedlings were then transferred into a growth chamber under identical conditions, a daily day/night regime of 14 h light (22,000 lx) at 17 °C and 10 h of darkness at 10 °C with 70% RH. The host responses to the stripe rust were recorded at least twice, approximately 18–21 days after inoculation, when the rust had fully developed on the susceptible control Mingxian 169. The disease resistance response was recorded by IT using the methods described above for adult plants (Li and Zeng 2002).

## Data analysis

The population structure (*Q* matrix) was analyzed using STRUCTURE software (version 2.3.4), with a burn-in length of 10,000 and 10,000 Markov chain Monte Carlo iterations for each *k*. Ten independent runs were carried out for each value of *k*. The maximum likelihood of each *k* value, the variance between the 10 runs, and the pedigree information of each line were weighted so that we could determine the optimal number of groups. The relative kinship (*K*) matrix was calculated using TASSEL 5.0.

Linkage disequilibrium (LD) was identified using TASSEL 5.0 software. The allele frequency correlations (*r*^2^) were calculated using the LD function according to Weir (1996). The significance of pair-wise LDs (*P* values) was computed using 1000 permutations. The LDs were calculated separately for loci on the same chromosome (intra-chromosomal pairs) and unlinked loci (inter-chromosomal pairs).

GWAS was carried out separately for two *Pst* races and four field environments to identify regions of chromosomes containing *Pst*-resistance genes, by using a mixed linear model (MLM), including both a *Q* matrix for fixed effects and a *K* matrix for random effects. The observed and expected *P* values for each trait (*Q*-*Q* plot) were used to compare the models and select the best model. Associations between SNP markers and stripe rust severity were evaluated using FarmCPU (fixed and random model circulating probability unification), an R package for GWAS, and genome prediction (http://zzlab.net/FarmCPU/index.html) (Liu et al. 2016). A threshold for significant associations between SNP markers and traits was set at 4.69 (−Log(0.5/total marker number)). The genetic positions (cM) of the SNP markers on the chromosomes were determined based on the 2015 wheat consensus map (Wang et al. 2014). The marker-trait associations were cross-referenced against all reported QTLs in the literature and the GrainGenes database (https://wheat.pw.usda.gov/GG3/) (Chao et al. 2007). The correlation between named *Yr* genes and significant SNP markers is analyzed using the Pearson method in SPSS v20.

## Results

### Evaluation of stripe rust resistance of wheat accessions

**Fig. 1:**
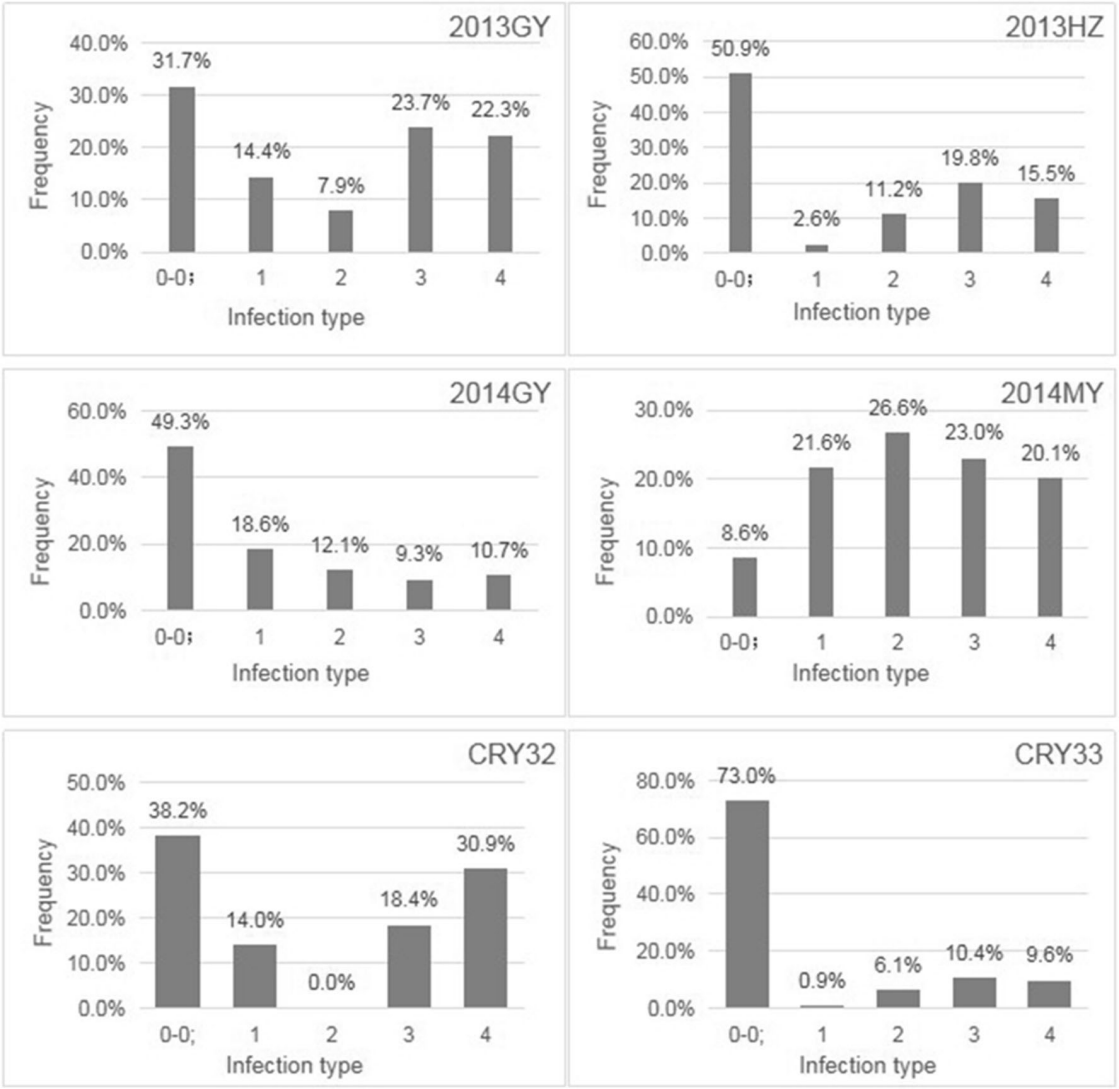
Evaluation of stripe rust resistance in 120 wheat accessions at different trials. 2013 GY and 2013 HZ indicate the sites of Guiyang and Hezhang in 2013, respectively, and 2014 GY and 2014 MY indicate the sites of Guiyang and Mianyang in 2014, respectively. CYR32 and CYR33 are two stripe rust races which are epidemical in Southwest China at the seedling stage

According to natural disease infestation, 120 wheat accessions were evaluated for stripe rust resistance over 2 years in four different environments. The percentage of accessions with resistance differed among the environments (Fig. 1, Supplementary Table S1). The highest percentage of accessions (50.9%) that were immune or nearly immune to stripe rust was located in Hezhang in 2013, while the lowest (8.6%) was observed in Mianyang in 2014. Overall, the highest percentage of accessions (80.0%) with resistance to stripe rust was observed in Guiyang in 2014, while the lowest occurred in Mianyang in 2014 (56.8%) and Guiyang in 2013 (54%). More accessions were resistant to CRY33 (80.0%) during the seedling stage than to CRY32 (52.2%).

### SNP statistics, LD, and structure matrix

In total, 120 wheat accessions were genotyped using a 90K SNP iSelect assay. Finally, 42,001 SNPs with a call rate of > 0.8 and minor allele frequency (MAF) > 0.05 were selected and used for principal component analysis (PCA), linkage disequilibrium (LD), and GWAS (Table 1). The SNPs were not evenly distributed, with most markers being distributed on the B genome (16,669) and the least on the D genome (11,573). There were seven homologous groups; the largest number of SNPs was in group 2 (8081), and the fewest in group 4 (4076). Of the 21 chromosomes, 2B harbored the most markers (3329) while 4D contained the fewest markers (961). On average, there were 2000 SNPs on each chromosome.

**Table 1:** Distribution of single nucleotide polymorphisms (SNPs) on the whole wheat genome

Genome	Group 1	Group 2	Group 3	Group 4	Group 5	Group 6	Group 7	Total
A	1931	2443	1704	1698	1756	2145	2083	13,760
B	2111	3329	2644	1417	3156	2165	1847	16,669
D	1731	2309	1318	961	1733	1753	1767	11,572
Total	5773	8081	5666	4076	6645	6063	5697	42,001

**Fig. 2:**
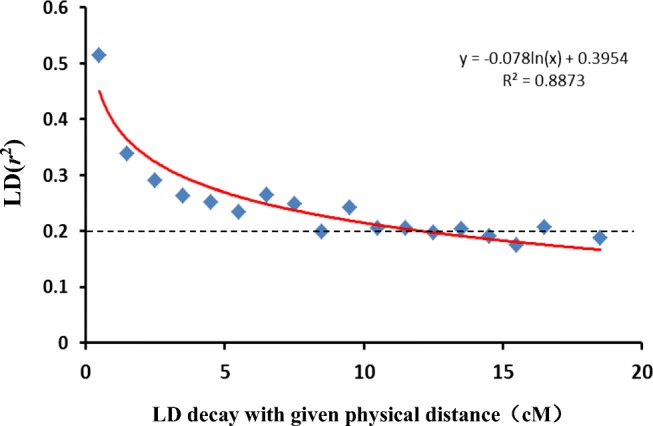
LD (*r*^2^) decay plot of marker pairs on all seven chromosomes as a function of genetic distance (in cM) for the 120 wheat accessions. The curve illustrates LD decay based on the nonlinear regression of *r*^2^ on genetic distance

We carried out LD analysis to study the wheat *Q* matrix in more detail and lay the foundations for GWAS. Intra-chromosomal LD was estimated using the squared allele frequency correlations (*r*^2^). For the whole genome, the average *r*^2^ was 0.22. For each chromosome, the highest value of *r*^2^ (0.32) was on chromosome 4D, while the lowest (0.13) was on chromosome 6D. At the intra-chromosomal level, the LD decay based on the nonlinear regression of *r*^2^ with respect to genetic distance was approximately 12 cM in our population (Fig. 2).

**Fig. 3:**
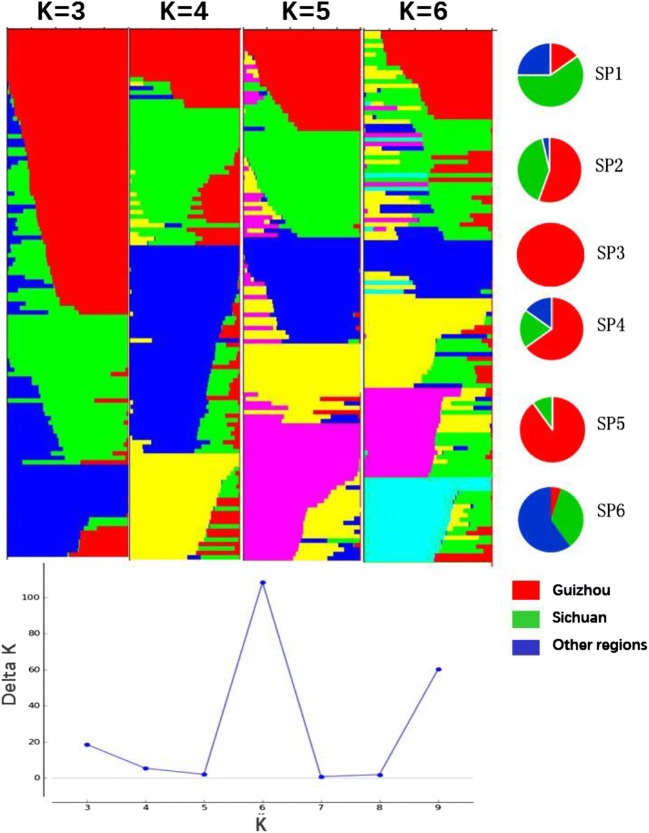
The population structure of 120 wheat accessions. Delta *K* is the function of *k* from the structure run, and the plateau at *k* = 6 indicates the number of sub-populations in our collection, clustering of wheat set genotypes into six sub-groups (SP1, SP2, SP3, SP4, SP5, and SP6). Six pie charts represent the proportion of varieties from different regions; red indicates Guizhou wheat varieties, green indicates Sichun’s, and blue indicates those in other regions

According to our structure analysis, the largest value of Δ*K* was observed at *K* = 6, which indicates that 6 is a suitable number of sub-populations (SP) (SP1-SP6) (Fig. 3). Accessions in SP1 (20), SP2 (27), and SP4 (20) were mainly derived from Guizhou and Sichuan, while those in SP3 (13) and SP5 (20) were mainly from Guizhou; 20 accessions in SP6 were from other regions.

### GWAS for resistance to wheat stripe rust

**Fig. 4:**
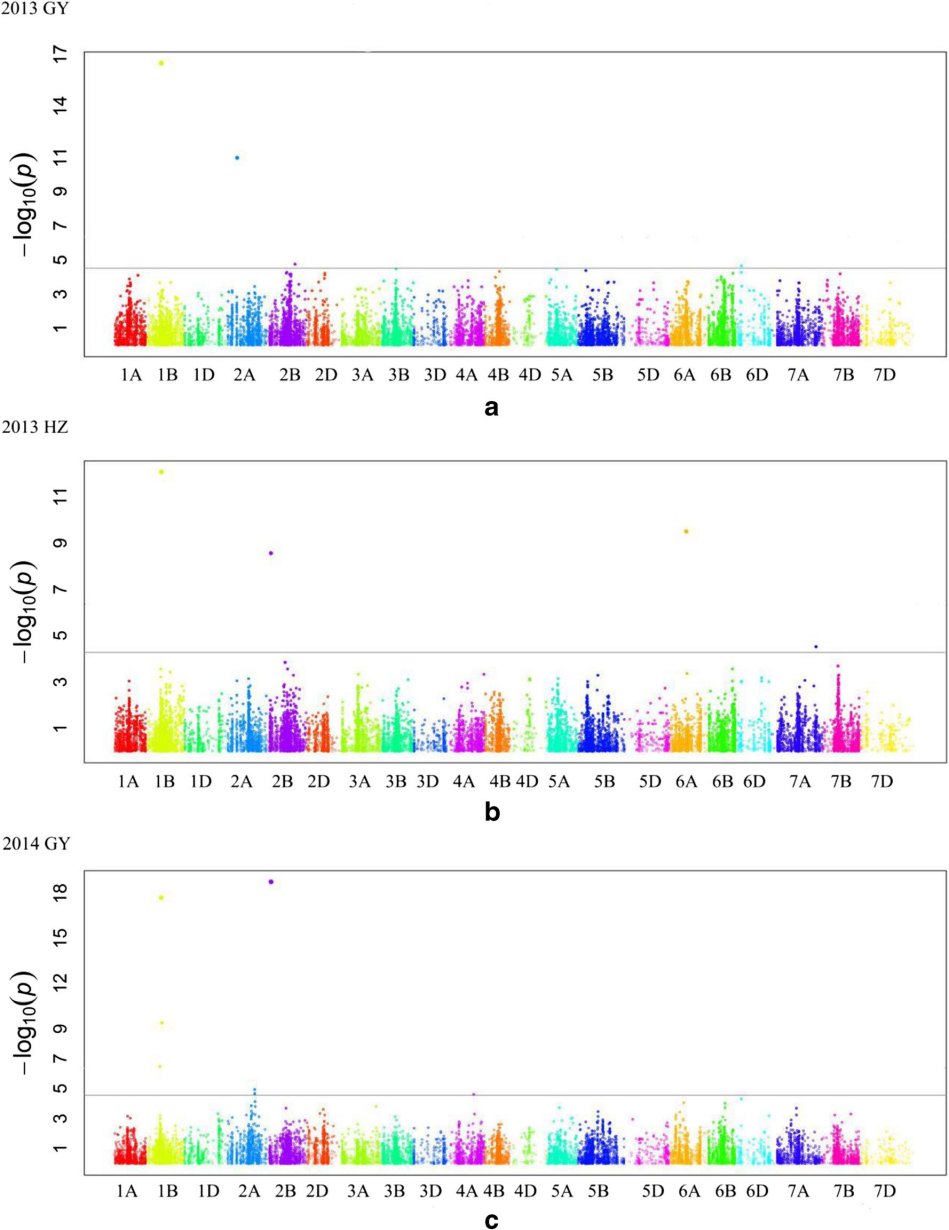

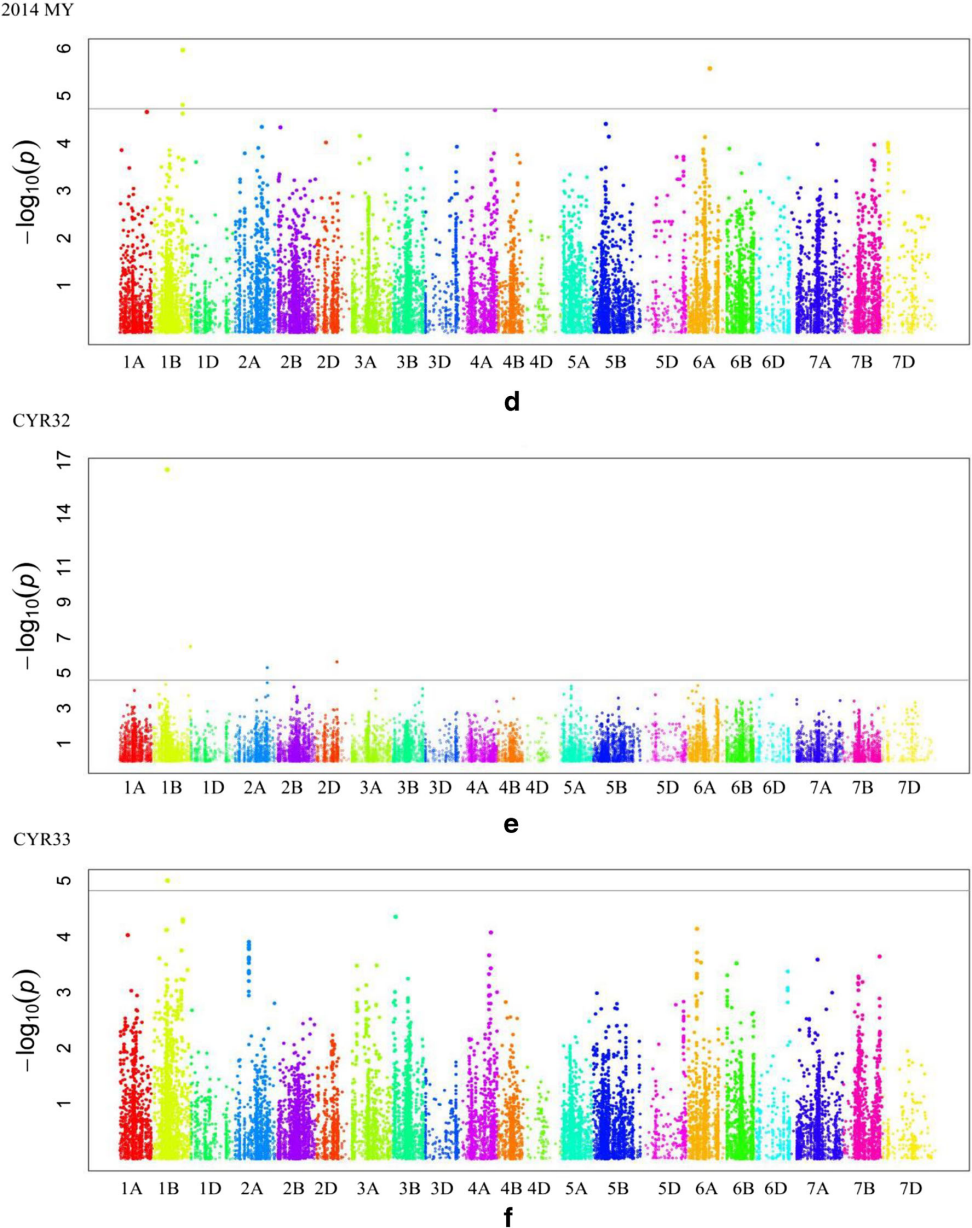
Genome-wide association study of wheat stripe rust resistance. **a**, **b**, **c** and **d** indicate Manhattan plots for adult plant resistance to stripe rust at sites of Guiyang and Hezhang in 2013, as well as Guiyang and Mianyang in 2014, respectively. **e** and **f** indicate Manhattan plots for seeding stage resistance to *Pst* races of CRY32 and CRY33, respectively

When the threshold −lg(*p*) was 4.69, a total of 16 SNPs were found to be significantly associated with the *Pst* resistance and were mapped to 11 regions of six chromosomes (Table 2, Fig. 4).

**Table 2:** Significant SNPs associated with wheat stripe rust resistance were evaluated in greenhouse experiments with two *Pst* races (CRY32 and CRY33 at seedling) and in field experiments (2013 GY, 2013 HZ, 2014 GY, and 2014 MY) for adult plant resistance. “Null” denotes no values of *r*^2^ could be obtained when using data of disease resistance in seeding stage

Marker name	Chrom	Position (Cm)	CRY32		CRY33		2013GY		2013HZ		2014GY		2014MY		MAF	SNP
			*P* value	*r*^2^	*P* value	*r*^2^	*P* value	*r*^2^	*P* value	*r*^2^	*P* value	*r*^2^	*P* value	*r*^2^		
BS00067586_51	1B	60.62									3.28E−07	0.48			0.12	T/C
Excalibur_c18876_334	1B	64.89	3.41E−17	Null			3.20E−17	0.54	8.78E−13	0.48	1.94E−18	0.50			0.48	T/C
RAC875_c5227_1385	1B	66.07			9.95E−06	Null									0.25	A/G
wsnp_CAP11_c631_419930	1B	69.30									4.09E−10	0.48			0.18	T/C
Tdurum_contig29087_628	1B	135.96											1.10E−06	0.49	0.25	A/G
Tdurum_contig29087_757	1B	135.96											1.55E−05	0.47	0.27	A/G
Excalibur_c25640_110	1B	173.62	3.07E−07	Null											0.48	T/C
BS00086365_51	2A	47.22					1.08E−11	0.51							0.25	A/G
RAC875_rep_c69619_78	2A	130.89									1.14E−05	0.47			0.34	A/C
IAAV7287	2A	151.45	5.01E−06	Null											0.07	T/C
Kukri_c55951_97	2B	19.16							2.74E−09	0.45	1.70E−19	0.57			0.40	T/C
BobWhite_c32319_313	2B	134.46					1.72E−05	0.46							0.41	A/G
BS00009637_51	2D	96.88	2.37E−06	Null											0.17	C/G
TA002369-0369	4A	154.30											1.99E−05	0.45	0.21	A/G
BS00079942_51	6A	71.24							3.19E−10	0.50					0.12	A/G
wsnp_Ex_c965_1846161	6A	100.12											2.67E−06	0.48	0.10	T/C

SNPs on chromosomes 1BL and 2BS were found to be significantly associated with *Pst* resistance in at least two experiments. The other nine chromosome regions were significantly associated with *Pst* resistance in only one experiment. On chromosome 1BL, a SNP (Excalibur_c18876_334) mapping on 64.89 cM was significant in the greenhouse experiment (CRY32) and the three field trials (2013GY, 2013HZ, 014GY). One SNP (RAC875_c5227_1385, 66.07 cM) was significant in a greenhouse experiment (CRY33) and two SNPs (BS00067586_51, 60.62 cM, and wsnp_CAP11_c631_419930, 69.30 cM) were significant in the 2014GY field trial. The three SNPs were located within 5 cM of the SNP (Excalibur_c18876_334) and were most likely associated with a gene that was predicted to significantly affect *Pst* resistance in all experiments, except for that at the 2014MY site. A SNP on chromosome 2BS (Kukri_c55951_97) was found to be significant in two field trials (2014GY and 2014MY) and was mapped to the chromosome position of 19.6 cM.

Of the other nine chromosome regions that were significantly associated with *Pst* resistance in a single experiment, two SNPs, Tdurum_contig29087_628 and Tdurum_contig29087_757, which were mapped to 135.96 cM on chromosome 1BL, were significant in the 2014MY field trial. Another significant SNP (Excalibur_c25640_110, 173.62 cM) was mapped 28 cM away from the two SNPs mentioned above on the same chromosome. Three significant SNPs, BS00086365_51 (47.2 cM), RAC875_rep_c69619_78 (130.89 cM) and IAAV7287 (151.45 cM), were detected on chromosome 2A in two trials (2013GY and 2014GY) and in the greenhouse experiment (CRY32). On chromosome 2B, one SNP (BobWhite_c32319_313), mapped to position 134.46 cM, was significant in the 2013GY field trial. One significant SNP (BS00009637_51) was detected on chromosome 2D at the seedling stage with CRY32 inoculation, while another significant SNP (TA002369-0369) was identified on chromosome 4A in the 2014MY trial. On chromosome 6A, two SNPs, BS00079942_51 (71.24 cM) and wsnp_Ex_c965_1846161 (100.12 cM), were found to be significant in the 2013HZ and 2014MY field trials.

### Alignments of significant resistance loci to known *Yr* genes

Correlation coefficients were calculated among the significant SNP loci obtained in above GWAS and the molecular markers of the known *Yr* genes. For the correlation among the significant SNP loci, several significant correlations were observed, of which two SNPs on chromosome 1B (Tdurum_contig29087_628 and Tdurum_contig29087_757) were significant positive correlations with *r* value of 0.97, and two SNPs on different chromosomes (BS00086365_51 on 2A and Kukri_c55951_97 on 2B) were significant negative correlations with *r* value of 0.93. For the correlation among the significant SNPs and the markers closely linked to known *Yr* genes, markers linked to *Yr15* (Barc8) and *Yr26* (We173) were significantly associated with SNPs (wsnp_CAP11_c631_419930, Excalibur_c18876_334, and RAC875_c5227_1385); of which, markers (We173) linked to *Yr26* and Excalibur_c18876_334 were significant positive correlations, with *r* value of 0.80 (Table 3**)**.

**Table 3:** Results of correlation analysis between marker linked to known genes and significant SNPs of GWAS

Markers	BS00067586_51	Excalibur_c18876_334	RAC875_c5227_1385	wsnp_CAP11_c631_419930	Tdurum_contig29087_628	Tdurum_contig29087_757	Excalibur_c25640_110	BS00086365_51	RAC875_rep_c69619_78	IAAV7287	Kukri_c55951_97	BobWhite_c32319_313	BS00009637_51	TA002369-0369	BS00079942_51	wsnp_Ex_c965_1846161	STS9/10	AF1/AF4	SC200	Barc8	SC372	We173
Excalibur_c18876_334(1B)	− 0.34**																					
RAC875_c5227_1385(1B)	− 0.32**	0.55**																				
wsnp_CAP11_c631_419930(1B)	− 0.15	− 0.18	− 0.01																			
Tdurum_contig29087_628(1B)	− 0.21*	0.31**	0.27**	0.10																		
Tdurum_contig29087_757(1B)	− 0.20*	0.33**	0.24*	0.10	0.97**																	
Excalibur_c25640_110(1B)	0.32**	− 0.56**	− 0.29**	0.13	− 0.22*	− 0.26**																
BS00086365_51(2A)	0.12	0.49**	0.34**	− 0.14	0.30**	0.28**	− 0.34**															
RAC875_rep_c69619_78(2A)	0.09	0.27**	0.35**	0.03	0.07	0.04	− 0.22*	0.22*														
IAAV7287(2A)	− 0.15	0.02	0.10	− 0.02	0.18*	0.06	− 0.01	0.14	− 0.04													
Kukri_c55951_97(2B)	− 0.10	− 0.43**	− 0.33**	0.18	− 0.27**	− 0.24**	0.32**	− 0.93**	− 0.14	− 0.14												
BobWhite_c32319_313(2B)	− 0.06	0.27**	0.08	− 0.10	− 0.18*	− 0.17	− 0.09	0.15	0.06	− 0.12	− 0.12											
BS00009637_51(2D)	0.10	0.10	0.07	0.13	− 0.02	0.01	0.01	0.01	0.14	− 0.51**	− 0.03	0.01										
TA002369-0369(4A)	0.03	− 0.18	− 0.31**	− 0.11	0.02	− 0.02	0.10	− 0.12	− 0.36**	0.17	0.03	0.08	− 0.10									
BS00079942_51(6A)	− 0.13	− 0.23*	− 0.09	− 0.02	0.13	0.07	0.20*	− 0.06	− 0.31**	0.35**	− 0.01	− 0.01	− 0.20*	0.36**								
wsnp_Ex_c965_1846161(6A)	− 0.07	0.19*	0.32**	0.15	− 0.11	− 0.10	− 0.12	.207*	0.33**	− 0.07	− 0.22*	0.00	0.12	− 0.53**	− 0.23*							
STS9/10(*Yr*5/2BL)	− 0.08	− 0.02	− 0.06	− 0.02	0.04	− 0.01	− 0.02	0.01	0.10	0.09	− 0.04	− 0.08	− 0.09	− 0.05	0.02	0.01						
AF1/AF4 (*Yr*9/1BL)	0.16	− 0.42**	− 0.35**	0.10	− 0.16	− 0.18	0.29**	− 0.29**	− 0.12	− 0.02	0.30**	− 0.07	− 0.11	0.10	0.04	− 0.20*	0.24**					
SC200 (*Yr*10/1BS)	0.26**	− 0.04	0.14	0.06	− 0.03	0.00	0.18	0.07	− 0.08	0.13	− 0.05	− 0.15	− 0.02	− 0.05	0.04	0.03	− 0.18	− 0.16				
Barc8 (*Yr*15/1BS)	− 0.12	− 0.18*	0.03	0.46**	0.01	0.04	0.07	− 0.23*	0.05	− 0.03	0.28**	− 0.12	− 0.02	− 0.12	− 0.13	0.12	0.02	− 0.07	0.12			
SC-372(*Yr*17/2AS)	− 0.16	− 0.19*	− 0.14	0.16	− 0.13	− 0.10	0.08	− 0.38**	− 0.03	0.09	0.41**	− 0.11	− 0.13	− 0.07	0.11	− 0.07	0.06	0.10	− 0.09	0.17		
We173 (*Yr*26/1BL)	− 0.32**	0.80**	0.48**	− 0.21*	0.26**	0.25**	− 0.59**	0.40**	0.31**	0.09	− 0.34**	0.28**	0.08	− 0.13	− 0.22*	0.19*	− 0.04	− 0.49**	− 0.10	− 0.17	− 0.24**	
Wmc44 (*Yr*29/1BL)	− 0.13	0.13	0.16	0.04	0.06	0.04	− 0.11	0.10	0.14	− 0.04	− 0.07	0.11	0.11	− 0.07	0.02	0.04	0.10	− 0.14	0.11	− 0.06	− 0.04	0.17

*Significantly correlated at the 0.05 level

**Significantly correlated at the 0.01 level

## Discussion

### Evaluation of stripe rust and resistance materials

For the virulence and frequency of wheat stripe rust races in Southwest China, the previous research showed that Sichuan region had the highest virulence, while Guizhou region had the lowest virulence (Zheng 2009). We observed similar results in this study. There were 31.7%, 49.3%, and 50.9% cultivars immune to local stripe rust races in the three environments in Guizhou (Guiyang in 2013 and 2014, Hezhang in 2013), but only 8.6% of the total cultivars exhibited immunity in Sichuan (Mianyang in 2014). The reason for this could be that a new *Pst* pathogen (V26) was first identified in Sichuan province in 2009, because it was virulent to wheat cultivars carrying the *Yr26* gene (Liu et al. 2012, 2015; Zhao et al. 2017). This race became the main virulent race in the epidemic in Mianyang, Sichuan, in 2014, while the prevailing races in Guizhou province at that time were still CRY32 and CRY33 (Chen et al. 2016a). In our collection, six lines (0308, Guinong18, Guinong19, Guinong28, TP3, and Guixie3) were bred in Guizhou province, which exhibited immunity to *Pst* races in all trials. As the V26 strain continues to spread, it is necessary to accelerate the use of these resistance sources in future breeding programs.

### *Q* matrix and LD analysis

According to the results of our analysis of the *Q* matrix, the optimal model was selected based on the observed and expected *P* values for each trait (*Q*-*Q* plots). The MLM model, which is based on *Q* and *K* matrices, proved to be a better fit and thus was selected for further association analysis (Supplementary Fig.S2). We divided the 120 accessions into six sub-groups. However, the *Q* matrix can be subdivided into nine groups according to the *K* matrix heat map, or into three sub-groups on the basis of geo-relationships (Supplementary Fig.S1). This may be explained as follows: (1) The original sources of these materials are not clear, nor are they strictly classified according to their geography, and could not be perfectly divided into three groups. (2) Most of these materials are characterized by similar genetics to those of the outer margin substances, such as the 6AS/6VL chromosome containing the *Pm21* gene, which is widespread in wheat in Southwest China, and existing stripe rust resistance genes, leading to unclear taxa.

The rate of decline of LD with distance depends strongly on mating patterns and populations. Because wheat is a strictly self-pollinating plant, LD decay is higher in wheat than in maize. The extent of LD in different wheat germplasms ranges from 1 to 40 cM, while those in maize (an outcrossing species) are within a few hundred bp to a few hundred kb (Tenaillon et al. 2001; Palaisa et al. 2004; Jung et al. 2004). In our germplasm, the genome-wide LD decay was estimated to be approximately 12 cM at *r*^2^ values of 0.2. Similar results have been reported in previous studies. For 482 wheat cultivars from the Australian Grains Genebank, the LD estimates ranged from 19.4 cM to 27.2 cM at an *r*^2^ value of 0.16 for cultivars from different periods (Joukhadar et al. 2017). Genome-wide *r*^2^ values declined rapidly to 0.2 within 10 cM among 43 US elite wheat cultivars and breeding lines (Chao et al. 2007). Owing to the low genetic diversity of populations, slow LD decay has been reported in several studies. For 200 ICARDA wheat genotypes, the LD started to decay at *r*^2^ values below 0.2, after 40 cM (Jighly et al. 2015). LDs with *r*^2^ = 0.2 extended to distances of up to 35 cM in 120 elite facultative/winter wheat genotypes from ICARDA (Tadesse et al. 2015). For 170 wheat lines derived from five CIMMYT elite spring wheat, LD declines by approximately 40 cM at *r*^2^ values of 0.2 (Crossa et al. 2007).

### Comparison between previous mapping genes/QTLs for stripe rust resistance

In this study, we identified a total of 16 SNPs associated with stripe rust resistance in our wheat accessions. These markers were located on eleven regions of chromosomes 1B, 2A, 2B, 2D, 4A, and 6A. All six chromosomes had been previously identified as containing stripe rust resistance genes (Maccaferri et al. 2015; McIntosh et al. 2017). Compared with the significant genes/QTLs identified in the current study to those reported in the literature, we found that several significant SNP loci have been mapped to similar positions of known *Pst*-resistance genes (Wang et al. 2008; Sun et al. 2010; Quan et al. 2013; Ren et al. 2012; Rosewarne et al. 2012; Maccaferri et al. 2015; Chen et al. 2016b).

One region (at about 65 cM) on chromosome 1BL was significantly associated with *Pst* resistance in greenhouse tests of the two *Pst* races (CRY32, CRY33) as well as natural infestation in three field trials (2013GY, 2014GY, 2013HZ). The SNP (Excalibur_c18876_334, 64.89 cM) was significant in the greenhouse experiment (CRY32) and the three environments, with an *r*^2^ value from 0.48 to 0.54. At present, two ASR genes have been reported on 1BL (*YrExp1* and *Yr26*) (Lin and Chen 2008; Wang et al. 2008), and only *Yr26* exhibits high resistance to races CRY32 and CRY33 (Zeng et al. 2015). The correlation analysis results showed that Pearson’s coefficient *r* value was 0.80 (at the significant level of 0.01) between the *Yr26* linked marker *we173* (1.4 cM) and the Excalibur_c18876_334, and we concluded that the SNP loci are most likely to closely link to gene *Yr26*. Another SNP (RAC875_c5227_1385) in this region also showed significant correlated with *we173* at *r* value of 0.48 and suggested that it may be linked to *Yr26* gene loci. In Mianyang, Sichuan province (2014MY), the prevailing *Pst* races were CRY32, CRY33, and V26; the two SNPs mentioned above were not detected because varieties carrying gene *Yr26* has lost resistance to the V26 race. However, another region on chromosome 1BL, including two SNPs (Tdurum_contig29087_628, Tdurum_contig29087_757) at the same position (135.96 cM), was significantly associated with *Pst* resistance in the 2014MY field trials. Two genes (*Yr29* and *YrExp2*) were reported on chromosome 1BL and confer APR to *Pst* races (William et al. 2003; Chen et al. 2014). *Yr29* is susceptible to race V26, while *YrExp1* exhibits resistance to V26 when combined with gene *YrExp2* (Zeng et al. 2015). Thus, the two SNPs may be linked to *YrExp1*. As this gene/QTL was detected in Mianyang in 2014, this result is highly relevant to wheat-resistant breeding in Southwestern China. This should be investigated in more detail in the future.

*Yr10* and *Yr15* were both mapped to chromosome 1BS in previous studies (Wang et al. 2002; Sun et al. 2010). However, *Yr10* and *Yr15* are rarely used in China. In 1980, only two lines of Chinese wheat landraces and foreign germplasms carried *Yr10* and none carried *Yr15* (Han et al. 2012). *Yr10* and *Yr15* were not detected in 75 Chinese commercial wheat cultivars (Zhang et al. 2014). Only 8% and 0% from Southwestern China carried *Yr10* and *Yr15*, respectively (Chen et al. 2016a). In our study, one SNP (BS00067586_51, 60.62 cM) on chromosome 1BS was found to be significantly associated with *Pst* resistance at Guiyang in 2014, which was in the vicinity of a SNP (Excalibur_c18876_334, 64.89 cM) closely linked to *Yr26*. According to the results of correlation analysis, SNP (BS00067586_51) was not linked to genes *Yr10* and *Yr15*, so it could be associated with a QTL for *Pst* resistance reported in this region (Quan et al. 2013; Ren et al. 2012; Rosewarne et al. 2012).

In this study, one SNP on chromosome 2A and two SNPs on chromosome 2B were significant at three sites (2013GY, 2013HZ, 2014GY). One very significant SNP (Kukri_c55951_97, 19.16 cM) on chromosome 2BS was identified at two sites (2013HZ, 2014GY), while SNP (BS00086365_51, 2A) was significant at the site 2013GY. Strangely, correlation analysis showed the two SNPs showed significantly negative association at *r* value of 0.93, so it was very likely that they were located at the same site. The known ASR *Yr* genes on chromosome 2BS had lost resistance to CYR32 and CYR33, and the two SNPs might be significantly associated with the QTLs for *Pst* resistance on this chromosome region (Rosewarne et al. 2008; Guo et al. 2008; Dedryver et al. 2009; Dolores et al. 2012). Another SNP (BobWhite_c32319_313, 134.46 cM) on chromosome 2BL was significant at Guiyang in 2013. On this chromosome, previous studies have shown that *Yr5* from *T. spelta* still exhibited high resistance against CYR32 and CRY33 (Macer 1966; Sun et al. 2002). However, *Yr5* was rarely used and almost undetectable in wheat cultivars and germplasms in China (Han et al. 2012; Zhang et al. 2014), and our correlation analysis also confirmed that SNP (BobWhite_c32319_313) did not show significant correlation with marker linked to *Yr5* gene. Moreover, *Yr3a* was also reported in the distal region of chromosome 2BL (Chen et al. 1996; Maccaferri et al. 2015), and exhibits resistance to the races CRY32, CRY33, and V26 when pyramiding with other *Yr* genes (Zeng et al. 2015). Therefore, SNP (BobWhite_c32319_313) is likely to be associated with gene *Yr3*, but further allelism tests will be required to confirm these results.

*Yr51* and some QTLs have previously been mapped to the long arm of chromosome 4A (Chen et al. 2012; Ramburan et al. 2004). In a similar region, SNP (TA002369-0369) was significantly associated with *Pst* resistance at 2014MY in this study, which could mean that it is linked to the genes or QTLs mentioned above. Additional allelism tests will be required to confirm these results.

Until now, no *Yr* genes were previously mapped onto chromosome 6AL, but some QTLs for *Pst* resistance were identified in this region (Lillemo et al. 2008; Prins et al. 2010; Lan et al. 2014). In this study, two SNPs on chromosome 6AL were significant at trials in Guiyang and Mianyang in 2014. SNP (BS00079942_51) was located at the position 71.24 cM at 2014GY, while SNP (wsnp_Ex_c965_1846161) was at the position of 100.12 cM at 2014MY. One of the two SNPs was significantly associated with a QTL for *Pst* resistance, which might correspond to the QTLs mentioned above.

The epidemic *Pst* races at three sites (2013GY, 2014GY, 2013HZ) were basically the same prior to 2009, mainly comprising CYR32 and CYR33, but those in Mianyang in Sichuan province have mostly included CRY32, CRY33, and V26 since 2010 (Liu et al. 2012, 2015; Chen et al. 2016a). Therefore, the loci detected in Mianyang were different from those detected at the other three sites in Guizhou province. In this study, four SNPs on three chromosomal regions were significantly associated with *Pst* resistance at Mianyang in 2014; these were located on chromosomes 1BL, 4AL, and 6AL. The four SNPs were likely to be associated with new QTLs/genes that confer resistance to race V26. Further studies will be required to confirm this hypothesis. *Yr61* on chromosome 7AS exhibited high ASR to currently prevalent *Pst* races in China (Zhou et al. 2014; Zeng et al. 2015), but none of the SNPs was detected on chromosome 7A in our germplasm. As the V26 race continues to spread, it is necessary to accelerate the use of effective genes (*Yr10*, *Yr15*, and *Yr61*) by using marker-assisted selection (MAS) in breeding programs. We also need to discover more resistance genes by transferring new genes from wild wheat relatives.

Our study demonstrates that most of Guizhou winter wheat accessions have high levels of stripe rust resistance, which could help to broaden the genetic resistance base of winter wheat breeding programs in southwestern China. For the accessions that we identified as carrying resistance to rust pathogens, further work is required to develop bi-parental populations of accessions to validate the resistance loci and develop user-friendly, tightly linked markers that can be employed to accelerate the incorporation of novel resistance into elite breeding wheat lines.

## Electronic supplementary material

ESM 1 (DOCX 604 kb)

ESM 2 (XLSX 35 kb)
