# Supplementary material for: Genome-wide association analysis of stripe rust resistance loci in wheat accessions from southwestern China
Source: J Appl Genet. 2020 Jan 7;61(1):37–50. doi: 10.1007/s13353-019-00533-8 (PMC6969011; doi:10.1007/s13353-019-00533-8)
Supplement: Supplementary file 1 — (DOCX 604 kb) [file 13353_2019_533_MOESM1_ESM.docx]

**SUPPLEMENTARY INFORMATION**

**Manuscript title**

Genome-wide association analysis of stripe rust resistance loci in wheat accessions from southwestern China

**Content**

**Supplementary Figure S1.** ....…….….…………………………….………………..........................2

**Supplementary Figure S2.** ....…….….…………………………….………………..........................3

**Supplementary Table S2.** ....…….….…………………………….………………..........................4

**Supplementary Figure S1.** A heatmap showing the kinship and phylogenetic relationships of 120 wheat landraces. The same phylogeny is shown in the left and above the heatmap. The red color represent strong relationships, while lighter colors indicate progressively weaker relationships.


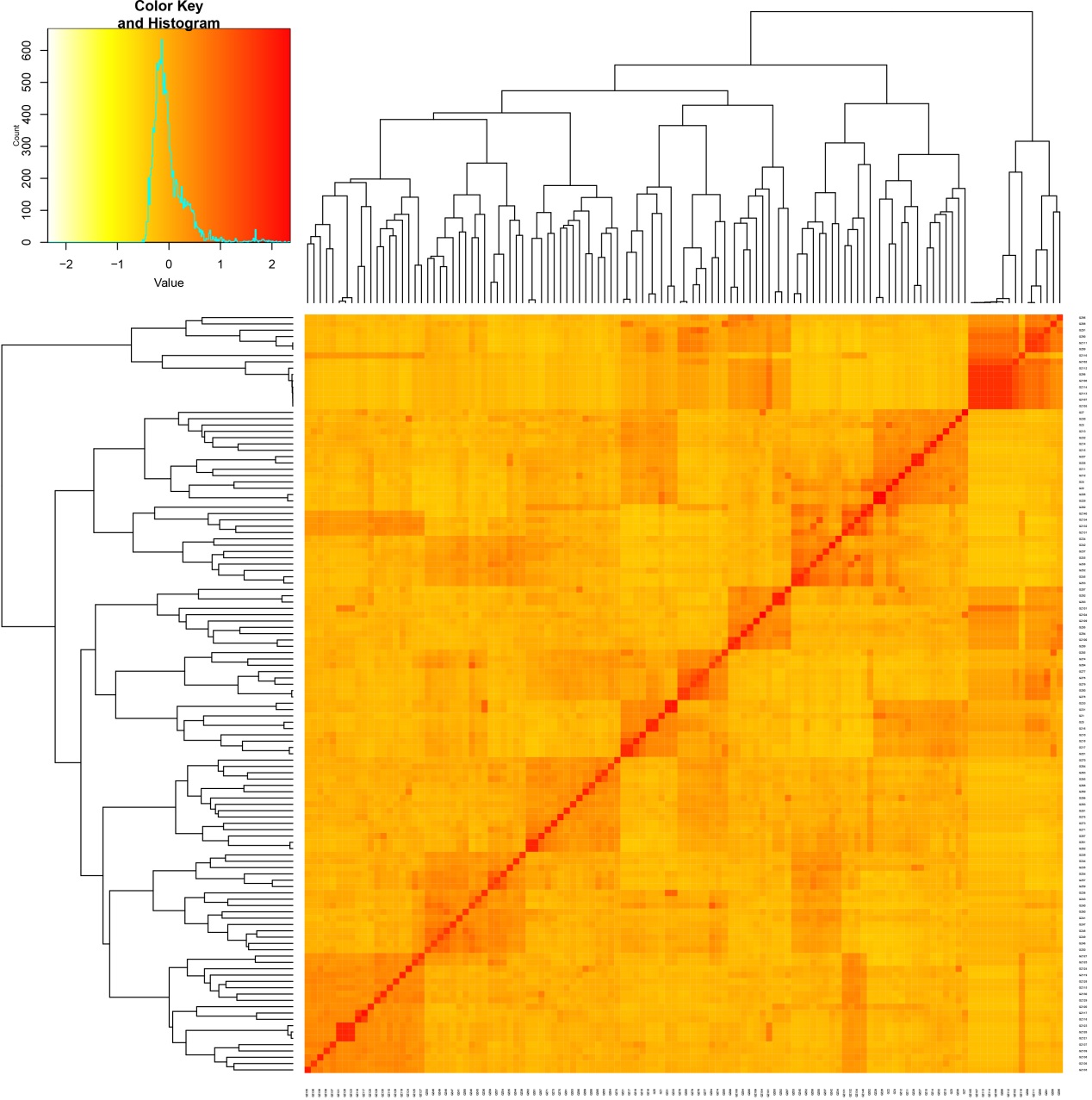


**Supplementary Figure S2.** Comparison between Q-Q plots of wheat stripe rust data from four field experiments (2013 GY, 2013 HZ, 2014 GY and 2014 MY) and two greenhouse experiments (CRY32, CRY33 seedlings).


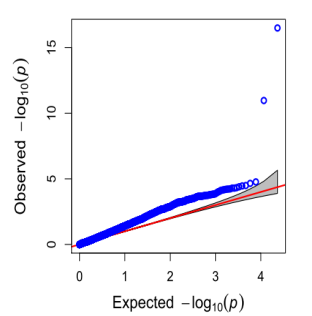


2013 GY


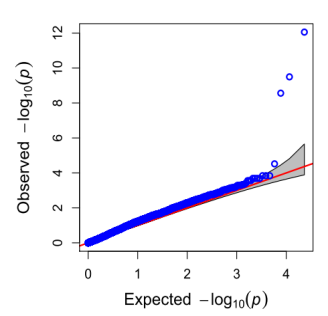


2013 HZ


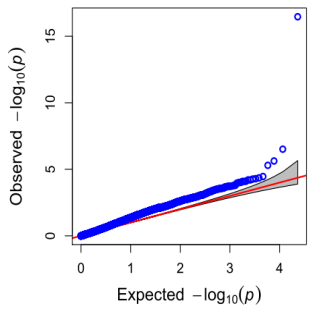

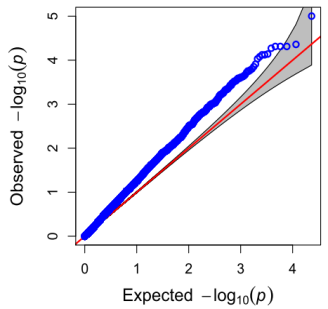

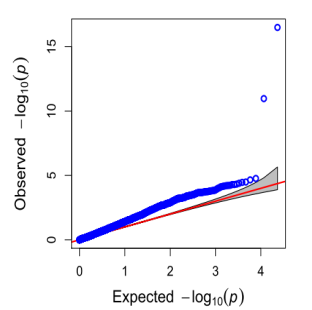


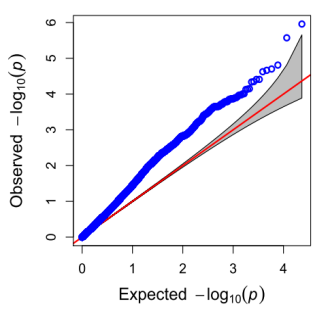


2014 MY

CRY33

CRY32

2014 GY

**Supplementary Table S2.** Molecular markers for stripe rust resistance genes

| ***Yr* Gene** | **Primer** | **Primer sequence (5’→3’)** | **Genetic distance (cM)** | **Amplicon length (bp)** | **Reference** |
| --- | --- | --- | --- | --- | --- |
| *Yr5* | STS9/10 | AAAGAATACTTTAATGAA | Completely linked | 289 | (Chen et al., 2003) |
|  |  | CAAACTTATCAGGATTAC |  |  |  |
| *Yr*9 | AF1/AF4 | GGAGACATCATGAAACATTTG | Rye specific | 1500 | (Francis et al., 1995) |
|  |  | CTGTTGTTGGGCAGAAAG |  |  |  |
| *Yr*10 | SC200 | CTGCAGAGTGACATCATACA | 0.5 | 200 | (Shao et al., 2001) |
|  |  | TCGAACTAGTAGATGCTGGC |  |  |  |
| *Yr*15 | Barc8 | GCGGGAATCATGCATAGGAAAACAGAA | linked | 576 | (Peng et al., 2000) |
|  |  | GCGGGGGCGAAACATACACATAAAAACA |  |  |  |
| *Yr17* | SC372 | ATG TCCGCCCTTCCACAACTC | Completely linked | 372 | (Jia et al., 2011) |
|  |  | CACTTGCCTATAAGCACAGAG |  |  |  |
| *Yr26* | We173 | GGGACAAGGGGAGTTGAAGC | 1.4 | 551 | (Wang et al., 2008) |
|  |  | GAGAGTTCCAAGCAGAACAC |  |  |  |
| *Yr*29 | Wmc44 | GGTCTTCTGGGCTTTGATCCTG | 5.6 | 242 | (William et al., 2003) |
|  |  | TGTTGCTAGGGACCCGTAGTGG |  |  |  |

The English in this document has been checked by at least two professional editors, both native speakers of English. For a certificate, please see:

http://www.textcheck.com/certificate/q9SIuQ
